# Primary Intracranial Melanoma with Early Leptomeningeal Spread: A Case Report and Treatment Options Available

**DOI:** 10.1155/2015/293802

**Published:** 2015-07-29

**Authors:** Rajesh Balakrishnan, Rokeya Porag, Dewan Shamsul Asif, A. M. Rejaus Satter, Md. Taufiq, Samson S. K. Gaddam

**Affiliations:** ^1^Department of Radiotherapy and Oncology, Square HospitalsLimited, Dhaka 1205, Bangladesh; ^2^Department of Neurosurgery, Square HospitalsLimited, Dhaka 1205, Bangladesh; ^3^Department of Pathology, Square HospitalsLimited, Dhaka 1205, Bangladesh

## Abstract

Primary CNS melanomas are rare and they constitute about 1% of all cases of melanomas and 0.07% of all brain tumors. These tumors are aggressive in nature and may metastasise to other organs. Till date less than 25 cases have been reported in the literature. The primary treatment for local intraparenchymal tumours is complete resection and/or radiotherapy and it is associated with good survival. However once there is disease spread to leptomeninges the overall median survival is around 10 weeks. In this case report we describe a primary intracranial melanoma without any dural attachment in 16-year-old boy who had radical excision of the tumor followed by radiotherapy who eventually had rapidly developed leptomeningeal disease and review the literature with a focus on the clinic pathological, radiological, and treatment options.

## 1. Introduction/Background

Primary CNS melanomas are rare and they constitute about 1% of all cases of melanomas and 0.07% of all brain tumors [1].

Till date less than 25 cases have been reported in the literature [2]. Malignant melanoma arises from either melanocytes or their precursor cells, melanoblasts [3]. As melanocytes are found in normal leptomeningeal tissue, it is not surprising that primary melanomas can grow within the central nervous system. In most instances, melanomas involving the CNS represent metastatic disease. Primary CNS melanomas are aggressive in nature and may metastasise to other organs. The primary treatment for local intraparenchymal tumours is complete resection and/or radiotherapy and it is associated with good survival. Temozolomide is an active drug which crosses the blood brain barrier and is known to be active in brain metastases from melanoma [4]. However once there is disease spread to leptomeninges the overall median survival is around 10 weeks [5]. In this case report we describe an adolescent male who had primary intracranial melanoma with early leptomeningeal disease progression and literature review with a focus on the pathological, radiological, and available treatment options.

## 2. Case Report

**Figure 1:**
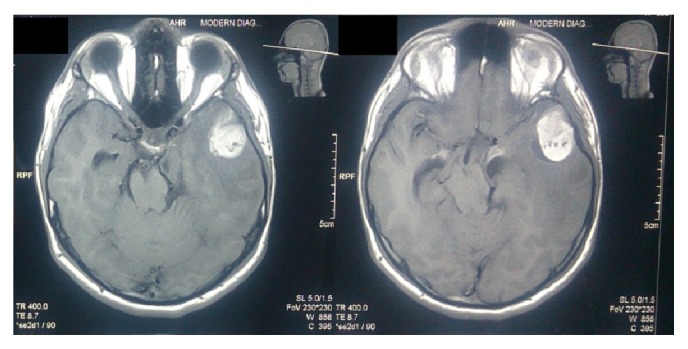
Preop MRI showing the lesion in the left temporal lobe with no dural attachment.

A 16-year-old boy presented with headache, convulsions, blurring of vision, and vertigo. On examination vitals were stable, fully conscious, and oriented, pupils 2 mm both equally reacting, no cranial nerve, motor and sensory deficit. His past medical history was unremarkable. He also had scattered melanocytic naevi over the back since birth without any itching or ulceration. None of the skin lesions were larger than 1 cm. He would fit into Fitzpatrick skin phototype IV. The patient had no family history of melanoma or atypical melanocytic nevus. Contrast enhanced CT brain revealed a heterogeneous high density mass at anterior part of the left temporal lobe with contrast enhancement. MRI brain demonstrated a T1 hyperintense and T2 hypointense mass in the left temporal lobe with perilesional oedema with no dural attachment (Figure 1). There was mild heterogeneous enhancement after administration of gadolinium.

**Figure 2:**
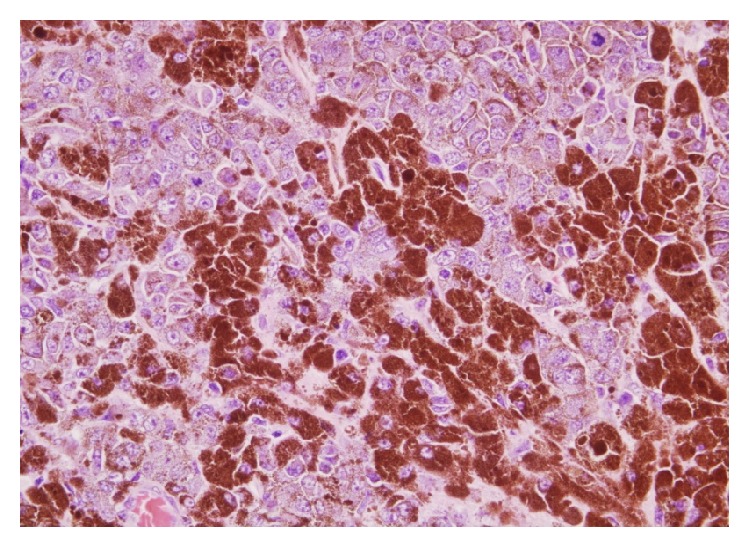
H&E stain, ×400, sheets of moderately large polygonal cells with round to oval pleomorphic nuclei, dispersed chromatin with mitotic activity (8–10/10 high power field).

**Figure 3:**
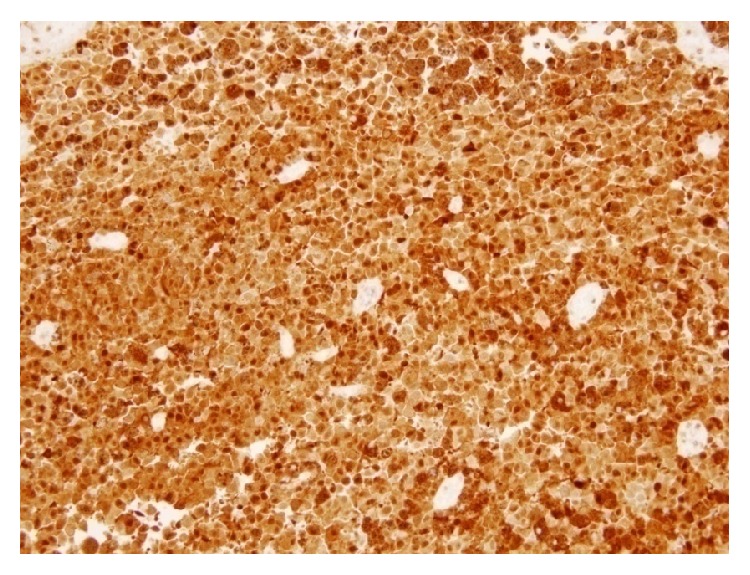
S100 staining, ×200, cells diffusely positive for S100.

The images prompted consideration of a hemorrhagic brain tumour. He had left temporal craniotomy and gross total excision of the tumor. Histopathological examination (HPE) revealed nervous tissue infiltrated by tumour composed of sheets of moderately large polygonal cells with round to oval pleomorphic nuclei, dispersed chromatin with mitotic activity (8–10/10 HPF). Numerous cells contained coarse, granular intracellular, intracytoplasmic, and extracytoplasmic brownish pigment resembling melanin. Periphery of the tumor showed tumor cells in the Virchow spaces of the brain tissue. On immunohistochemistry the tumor cells showed diffuse positivity for human melanin black-45 (HMB-45) antibody and S100 and weak patchy cytoplasmic positivity for antimelanosomal antibody MART-1 (Melan A) which were all suggestive of melanocytic origin (Figures 2 and 3). Tumor cells were negative for epithelial membrane antigen (EMA) and glial fibrillary acidic protein (GFAP) which effectively ruled out a meningioma or a glial neoplasm. HPE was reported as melanoma. He then underwent whole body PET-CT and it did not reveal any FDG avid regions except for small foci in rectal lumen. He had a colonoscopy which showed a rectal polyp. Biopsies from largest skin naevi found in the left gluteal region were reported as benign intradermal nevus and biopsy from the rectal polyp was reported as juvenile rectal polyp. His fundoscopy did not reveal any uveal melanoma. This suggested that we were dealing with a primary intracranial cerebral melanoma. Postoperative MRI brain showed no residual brain tumor.

**Figure 4:**
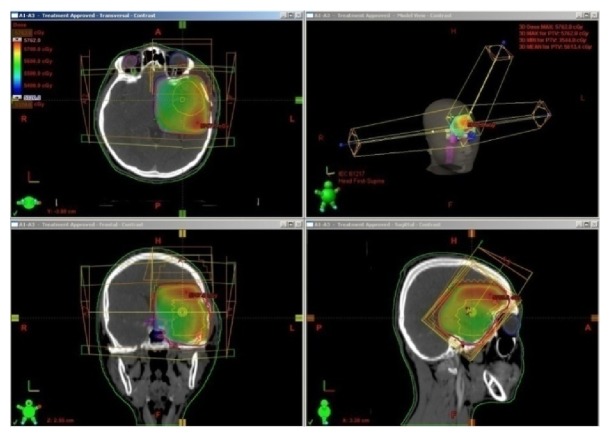
Radiotherapy was administered by 3D conformal technique and the dose wash shown in the figure.

**Figure 5:**
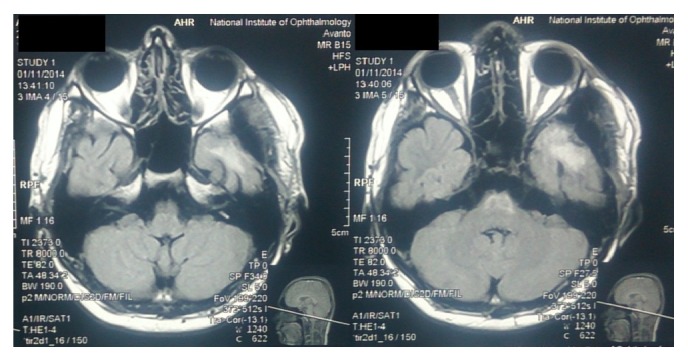
Post-ChemoRT images showing no recurrent/residual tumor.

**Figure 6:**
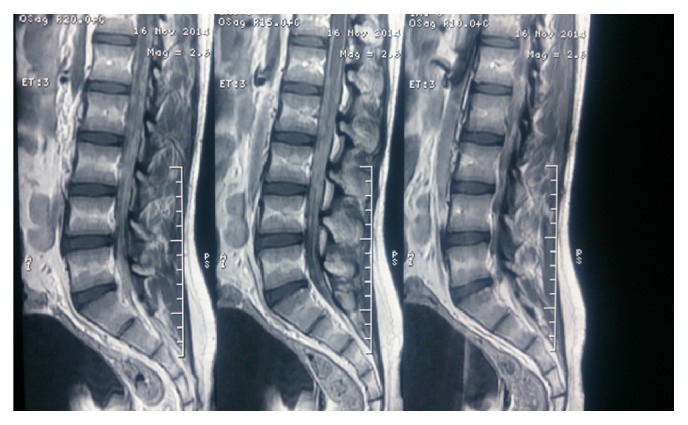
Spine MRI showing drop metastases in the lumbosacral spine.

**Figure 7:**
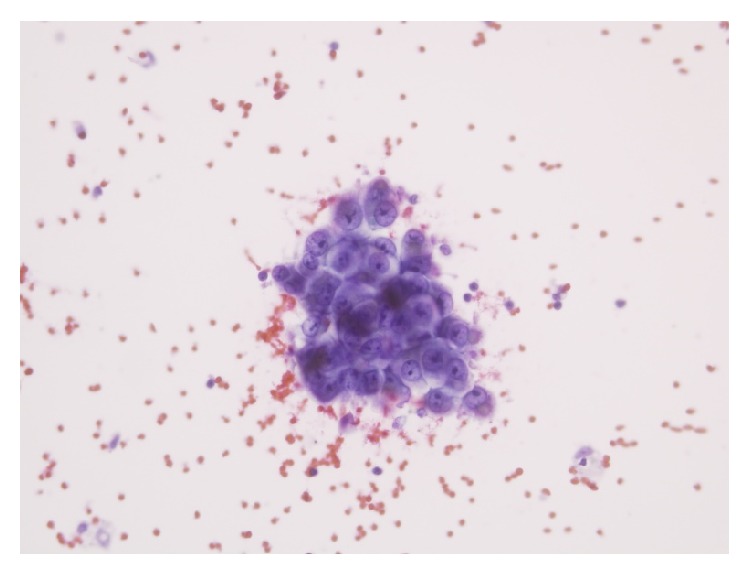
CSF cytospin smear showing atypical cells in small clusters with intracytoplasmic melanin pigment.

He then received adjuvant radiation (Involved Field 3DCRT) along with concurrent Temozolomide to a total dose of 5600 cGy in 28 fractions, 200 cGy per fraction, and five days in week to the postoperative tumor bed using 6 MV linear accelerator (Figure 4). Concurrent Temozolomide was given at a dose of 75 mg/sq·m to a dose of 100 mg for six and half weeks. His MRI brain done four weeks after completion of chemoRT showed no disease recurrence and hence he was started on adjuvant chemotherapy with Temozolomide (Figure 5). He presented one week after starting adjuvant chemotherapy with complaints of severe back pain which was not controlled with analgesics. He also progressed to have constipation and retention of urine. MRI whole spine screening showed multiple tiny T1 hyperintense and T2 hypointense intradural nodular lesions (Figure 6) along the intrathecal nerve roots along with diffuse enhancement of the lower dorsal cord and along the pons. His repeat metastatic workup did not show any lesions in the liver or lungs. His cerebrospinal fluid (CSF) cytospin done now showed melanoma cells (Figure 7) and he was started on Palliative RT to the lumbosacral region (3000 cGy in 10 fractions). He had marginal symptom relief and he had progression of the leptomeningeal disease. He developed cranial nerve deficits and the patient was not willing for any further treatment. He opted for best supportive care and he died from leptomeningeal disease progression at 7 months after diagnosis. The patient's family has consented for publication of his case and the signed form is available with the author.

## 3. Discussion

Melanocytes are of neural crest in origin and they migrate during development to the skin, eyes, oral cavity, and the leptomeninges. Primary melanoma of the CNS originates from the melanocytes of the leptomeninges. In children it occurs primarily in patients with neurocutaneous melanosis. In children the primary melanoma of the CNS is of poor prognosis unlike in adults and is associated with an overall survival of 8 months from the initial presentation [6].

### 3.1. Origin of Primary CNS Melanoma

Several histogenetic theories (mesodermal origin, ectodermal origin, and neurogenic origin) have been proposed regarding the origin of melanin cells [7]. The most recent theory is the implication of overexpression of oncogenic NRAS in melanocytes during embryonic development [8]. It is also interesting to note that NRAS mutations are the second most common mutations to occur in melanomas (20%) next to BRAF mutations (50%) [9].

### 3.2. Diagnosing Primary CNS Melanoma

It is difficult to diagnose a primary CNS melanoma upfront. Majority of patients with intracranial melanomas present with features of raised intracranial tension (43%), neurological deficits (35%), features of subarachnoid haemorrhage (14%), or convulsions (12%) [10]. There is a documented male predominance in primary CNS melanomas (same as our patient). It is usually a diagnosis of exclusion of metastatic disease from a skin or oral or uveal melanoma by proper evaluation as brain is the most common site for metastases from breast, lung, or melanoma. Hayward in his landmark publication had set down criteria to classify tumors into primary melanomas, secondaries, and melanin containing variants of other intracranial tumors. According to Hayward, A solitary cerebral lesion, intramedullary or leptomeningeal involvement, hydrocephalus, and pineal/pituitary tumors could be primary melanomas provided that no melanomatous lesions are found outside the CNS [11].

### 3.3. Neuroradiology

The preoperative diagnosis of primary CNS melanoma is difficult, except in cases associated with neurocutaneous melanosis [12] or when melanin or melanin-containing cells are detected in the cerebrospinal fluid [7]. The CT findings of intracranial melanomas, including high-density mass on precontrast scans, homogeneous enhancement, and marked peritumoral oedema, are not specific [13–15]. MRI brain with gadolinium is the imaging of choice for a patient with suspected CNS melanoma. The MRI findings of a CNS melanoma are so typical that they are hyperintense on T1 weighted images and hypointense on T2 weighted images due to presence of melanin [16]. The radiological differentials are haemorrhage, primary or metastatic malignant intracranial melanomas [17]. These lesions are hyperperfused on perfusion imaging and have an elevated choline and reduced NAA peak on MR spectroscopy. Melanocytomas and meningiomas usually appear isointense on T1WI and display homogenous enhancement. Both display dural attachment and occasionally brain invasion [18]. The differential diagnosis is more difficult in some cases of meningioma with cells containing melanin pigment, ectopic meningioma, or hemorrhagic meningioma [19]. As MRI of our patient shows T1WI hyperintensity and T2WI isointense to hyperintensity with peritumoral edema, our initial impression was a tumor haemorrhage either primary or secondary. In our patient, there was no dural attachment but there was extensive peritumoral edema which could be due to brain invasion. Peritumoral edema seen as T2 hyperintensity reflects the rapid growth rate of the tumor.

The role of FDG PET is also important as it would be of immense help to rule out the presence of disease anywhere else outside the CNS. The whole body FDG PET has specificity of about 91% in diagnosing locoregional disease and for staging of melanomas [20].

### 3.4. Immunohistochemistry

Primary melanocytic neoplasms of the central nervous system (CNS) consist of a broad spectrum ranging from well differentiated melanocytoma to its overtly malignant counterpart, melanoma [21]. HMB-45, MART-1 (Melan A), S-100, and tyrosinase are almost always diffusely positive in primary melanocytic neoplasms of the CNS. Melanomas do not stain for EMA and this is used to differentiate from meningiomas [22] Occasionally, in cases of neurilemmoma and pigmented malignant schwannoma, more rarely in ependymoma, gliosarcoma, and cerebral primitive neuroectodermal tumors there could be some focal positivity of HMB 45.

Immunohistochemistry results in our patient showed diffuse positivity for HMB-45 and S-100 protein and weak patchy cytoplasmic positivity for Melan A. Also tumor cells were negative for EMA and GFAP. Histopathology and immunohistochemistry of the patient's tumor led us to diagnosis of a malignant melanoma. We reached a diagnosis of primary CNS melanoma after excluding extracranial disease in the skin, uvea, or the mucosa along with the HPE and IHC from the specimen. He fulfilled the Hayward criteria for diagnosing a primary CNS melanoma. BRAF mutations analysis could not be done in this patient as our lab was not equipped with the facilities for doing it. Moreover BRAF inhibitors are not available in our country for patient use.

### 3.5. Treatment Options

There are no specific guidelines to our knowledge for the management of the primary CNS melanomas as they are a rare tumor. There are no published randomised trials which would help us in the management of these tumours.

Complete excision of the tumor is the mainstay of the management and it helps to reduce the raised ICT. Primary CNS melanocytic tumours have better outcome by surgical intervention, with or without additional adjuvant treatment [23, 24]. Radiotherapy and chemotherapy play an important role after surgery even though it has been considered that melanomas are one of the radioresistant or chemoresistant tumours.

Radiation can be either given whole brain RT or involved field RT with margins with or without concurrent chemotherapy with Temozolomide. The agents that have been tried with RT include BRAF inhibitors, intraventricular chemotherapy, intrathecal Methotrexate+ steroids, and intrathecal recombinant Interleukin-2.

There are reports where RT up to 5400 Gy has been tried to the postoperative tumor bed in conventional fractionation successfully in the adjuvant setting [25].

Involved field radiotherapy at the symptomatic regions or for the bulky disease is component of treatment for patients with leptomeningeal spread. When a patient presents with cranial nerve involvement, RT helps to reestablish the normal CSF flow and these patients have hydrocephalus [26].

IntraCSF chemotherapy has been tried for these tumours with leptomeningeal disease by a few researchers. The primary intent was that since most chemotherapy agents do not cross the BBB they attempted to directly instil the chemotherapy in the CSF to kill the cells directly. Le Rhun et al. had used Methotrexate (15 mg) and Dexamethasone (5 mg) once or twice weekly [27].

Lee et al. had treated patients with advanced melanoma with whole brain RT (30 Gy in 10 fractions) followed by BRAF inhibitors like Vemurafenib [28] and had reported long-term stabilisation of the Leptomeningeal disease for 18 months.

WBRT (40 Gy in 20 fractions) along with concurrent intrathecal Methotrexate (15 mg once weekly for 8 weeks) has been tried and it had resulted in a progression free interval of 8 months [26].

In a retrospective study in advanced malignant melanomas done by Paul et al., he demonstrated significant reduction in CNS relapse in the Temozolomide group compared to DTIC at 19-month follow-up. In view of these results and as the lineage of primary CNS melanomas is similar to melanomas, TMZ could play an important role in the concurrent and adjuvant treatment schema and this needs to be evaluated further [4].

**Figure 8:**
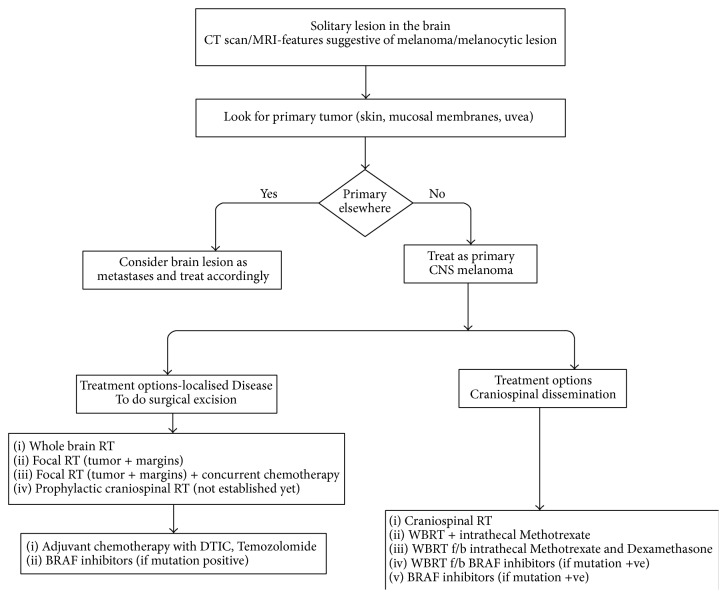
Proposed diagnostic/treatment algorithm for primary CNS melanoma.

The role of craniospinal irradiation is not yet defined in treating patients with intracranial CNS melanoma as they frequently metastasize to the leptomeninges (LM) in the due course of disease. The benefits and the risks of subjecting the patient to the prophylactic craniospinal axis irradiation need to be weighed before such a treatment is being offered to the patient in patients who do not have leptomeningeal dissemination at presentation. However in case of patient presenting with LM dissemination they could be considered for craniospinal RT [29]. A diagnostic and treatment pathway for these rare tumors is depicted in Figure 8.

## 4. Conclusion

It is difficult to diagnose primary CNS melanoma in the absence of any cutaneous melanosis. A high index of clinical suspicion along with good pathology reporting is the key in diagnosing these extremely rare tumours. PET CT whole body helps to rule out the presence of occult primary anywhere else. It is necessary to differentiate the benign melanocytomas from the malignant melanomas. The preferred treatment option is total surgical excision if feasible followed by postoperative adjuvant therapy with radiation and chemotherapy. The role of BRAF inhibitors, immunomodulating therapies, prophylactic craniospinal irradiation, and intrathecal chemotherapy needs to be evaluated in future.

## Abbreviations

3DCRT: 3-dimensional conformal radiotherapy
BBB: Blood brain barrier
CECT: Contrast enhanced computerised tomography
CSF: Cerebrospinal fluid
CNS: Central nervous system
LM: Leptomeningeal
PET-CT: Positron emission tomography-computerised tomography
RT: Radiotherapy
WBRT: Whole brain radiotherapy.
